# Bile duct infestation with Enterobius vermicularis diagnosed after cholecystectomy: About two case reports

**DOI:** 10.1002/ccr3.5038

**Published:** 2021-11-06

**Authors:** Salwa Nechi, Ghada Gharbi, Amel Douggaz, Malak Boughdir, Abir Chaabane, Mohamed Karim Mfarrej, Emna Chelbi

**Affiliations:** ^1^ Department of Pathology Mohamed Taher Maamouri Hospital Nabeul Tunisia; ^2^ Faculty of Medicine of Tunis University of Tunis El Manar Nabeul Tunisia; ^3^ Department of Gastroenterology Mohamed Taher Maamouri Hospital Nabeul Tunisia; ^4^ Department of surgery Mohamed Taher Maamouri Hospital Nabeul Tunisia

**Keywords:** bile duct infestation, cholecystectomy, Enterobius vermicularis

## Abstract

Intestinal infestation with Enterobius vermicularis is common, especially in the developing countries. However, its migration in the bile ducts is rare, often diagnosed after cholecystectomy. More investigations are needed to define its involvement in symptoms presented by patients and the likelihood of complications.

## Graphical abstract

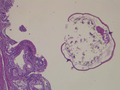

## 1 INTRODUCTION

Parasitic infestations are frequent, especially in the developing countries. The intestines are the most common sites of involvement by the parasites but biliary tract infestation can also be observed with different complications contributing to the morbidity and mortality caused by the disease.^1^ Ascaris lumbricoides is the most common parasite of the intestinal tract, which can migrate into the bile ducts.^2^ Other parasites have also been involved. Enterobius vermicularis is a rare cause of biliary tract infestation, and only one case has been reported in the literature.^3^ We report two cases of a biliary tract infestation with pinworm diagnosed after cholecystectomy.

## 2 METHODS

We report two cases of a biliary tract infestation with pinworm diagnosed after cholecystectomy.

## 3 CASE REPORTS

### 3.1 Case 1

**FIGURE 1:**
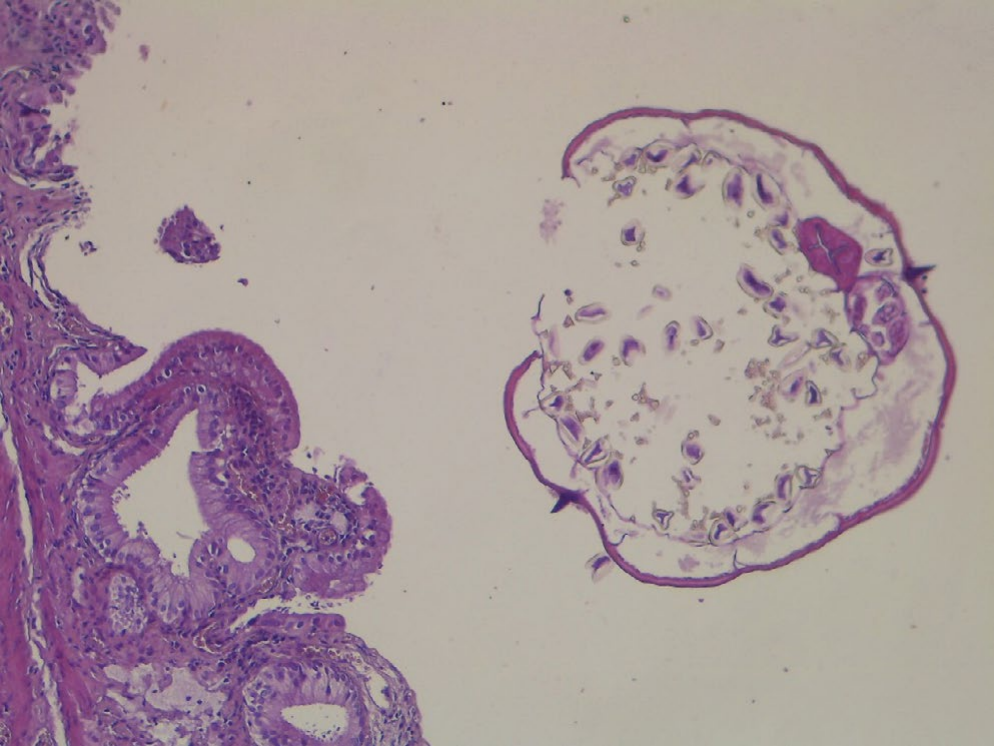
Enterobius vermicularis in the gallbladder lumen with cuticle and two lateral spurs

A 60‐year‐old female patient from a rural area, with a history of diabetes mellitus, presented with a 12‐month history of intermittent biliary colic. Physical examination and blood tests were normal. An abdominal ultrasound showed a thin‐walled gallbladder with multiple small gallstones filling the lumen. The patient was operated on. She had a laparoscopic cholecystectomy. At surgery, stones were found in the gallbladder and the cystic duct. Pathological examination of the gallbladder revealed an altered gallbladder wall by chronic cholecystitis lesions associated with cholesterolosis. The presence of a pinworm section at the vesicular lumen was also noted (Figure 1).

### 3.2 Case 2

**FIGURE 2:**
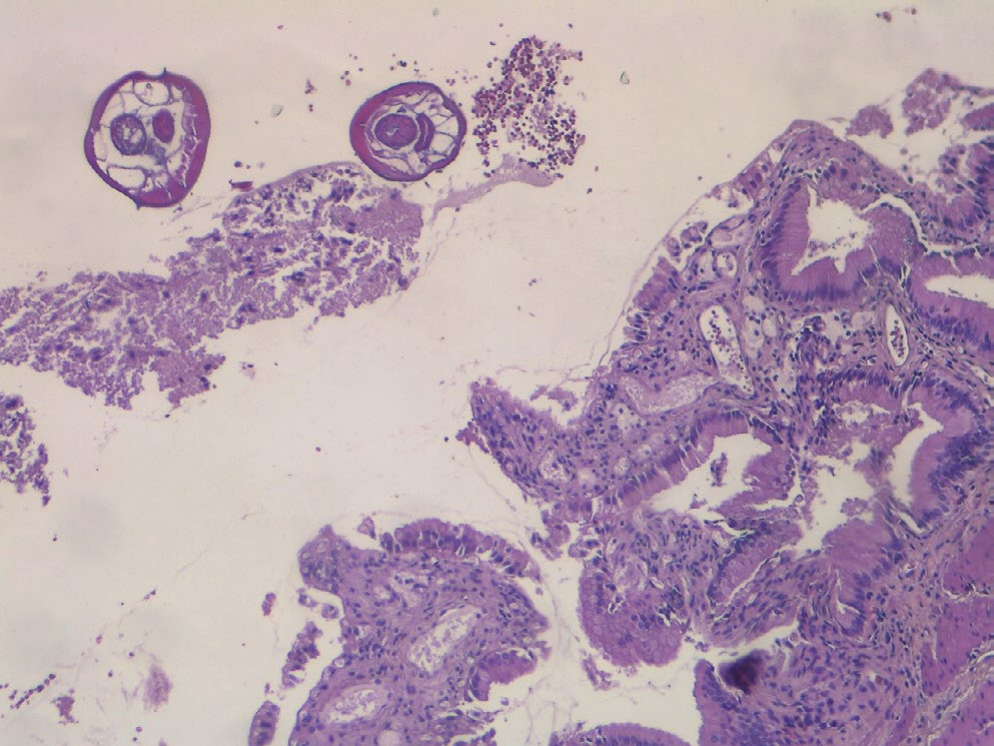
Enterobius vermicularis in the gallbladder lumen

A 25‐year‐old female patient from a rural area, with no medical history consulted the emergency department suffering from right hypochondrium pain accompanied by nausea and vomiting. Physical examination revealed tenderness of the right hypochondrium and blood tests were normal. An abdominal ultrasound showed a gallbladder sludge without signs of complications. The patient was operated on. She had a laparoscopic cholecystectomy. Pathological examination of the gallbladder revealed an altered gallbladder wall by chronic cholecystitis lesions associated with cholesterolosis and vesicular pinworm (Figure 2).

## 4 DISCUSSION

Parasitic disease of the biliary tract is always asymptomatic, and it can be caused by many trematodes residing in the biliary tree such as Clonorchis sinensis, Opisthorchis viverrini, Opisthorchis felineus, Fascioliasis, and Dicrocoelium dendriticum.^1^, ^4^ Ascaris lumbricoides, which is the most common parasite of the intestinal tract, can also be involved after evading the papilla.^1^ These organisms can cause many complications such as intrahepatic stones, recurrent pyogenic cholangitis, cirrhosis, cholelithiasis, pancreatitis and cholangiocarcinoma.^1^ Enterobius vermicularis is another intestinal parasite which can migrate into the biliary tract.

Enterobius vermicularis is a common intestinal nematode that is common all over the world, especially in temperate regions, where fecal sanitation is poor.^5^ The most typical symptoms of pinworm infestation are pruritus of the perianal area, particularly at night, Insomnia, restlessness, enuresis and irritability.

Enterobius vermicularis has a simple direct life cycle, which takes place in the gastrointestinal lumen. Infection occurs via oral ingestion of infective eggs. Once in the digestive system of the host organism, the external membrane of the eggs softens. After passing through the pylorus, the pinworm larvae hatch in the small intestine. After molting twice, the worms copulate and then migrate downwards to the large intestine, where they can be found in large numbers particularly in the cecum, appendix or ascending colon.^6^ Very rarely, incidental involvement of other organs occurs (<1% of cases),^6^ it can involve the retrocecal tissues, the peritoneum,^7^ the genital female and male tract^8^, ^9^, ^10^ and less frequently the liver.^11^ There are isolated case reports of infection involving the salivary glands,^12^ nasal mucosa, eye,^13^ skin,^14^ and lungs,^15^ presumably due to autoinoculation of these sites with eggs or adult worms from the intestinal tract.^13^

Only one case reporting Enterobius vermicularis migration into the biliary tract has been reported in the literature in an older woman.^3^ As it was the case of the two patients reported above, she was operated on for Cholecystocholelithiasis. Intraoperatively, live helminths consistent with Enterobius vermicularis were found on the extracted stones from the cystic duct.

Thus, biliary involvement is uncommon in enterobiasis, the clinical manifestations are non‐specific and there are no specific signs in imaging findings. Its involvement in the etiology of symptoms presented by patients and the likelihood of complications still unknown and need more investigations.

## 5 CONCLUSION

Enterobius vermicularis infestation of the biliary tract is an uncommon location of this intestinal parasite. It is usually an accidental diagnosis during surgery or on the pathological data of gallbladder after cholecystectomy.

## CONFLICT OF INTEREST

None declared.

## AUTHOR CONTRIBUTIONS

SN and GG: wrote the paper. GG: reviews the literature. SN, AD, AC, and MKM: contributes by the pathology pictures as well as the interpretation of figures. MB: operated the patient on. EC: The head of the pathology department at the Mohamed Taher Maamouri Hospital.

## CONSENT

Patient personal data have been respected.

## Data Availability

No data are available for this submission.
